# Optimization of red teff flour, malted soybean flour, and papaya fruit powder blending ratios for better nutritional quality and sensory acceptability of porridge

**DOI:** 10.1002/fsn3.624

**Published:** 2018-03-25

**Authors:** Kiros Mezgebo, Tefera Belachew, Neela Satheesh

**Affiliations:** ^1^ Department of Postharvest Management College of Agriculture and Veterinary Medicine Jimma University Jimma Ethiopia; ^2^ Department of Food Science and Postharvest Technology College of Agriculture and Environmental Sciences Adigrat University Adigrat Ethiopia; ^3^ Department of Human Nutrition College of Health Science Jimma University Jimma Ethiopia; ^4^ School of Chemical and Food Engineering Institute of Technology Bahir Dar University Bahir Dar Ethiopia

**Keywords:** β‐carotene, complimentary foods, D‐optimal mixture design, papaya fruit powder, porridge, proximate composition

## Abstract

This study was carried out to optimize the compositions of red teff flour with malted soybean flour and papaya fruit powders for better nutritional quality and sensory acceptability of porridge. Total eleven formulations of the composite flours were determined using D‐optimal mixture design with the help of Minitab Version 16 Statistical Software. The three ingredients were considered in the ranges of 55%–70%, 20%–30%, 5%–15% for red teff flour, malted soybean flour, and Papaya fruit powder, respectively. The prepared porridge samples from formulations were analyzed for nutritional composition, antinutritional factors, and sensory acceptability. Results of the study showed the significant difference (*p* < .05) in ash, fat, fiber, protein, carbohydrate, energy, iron, calcium, zinc, β‐carotene, phytates, tannin, appearance, taste, mouthfeel, and overall acceptability as the composition of ingredients were changed. The overall optimum point was found in a range of red teff flour (60%–70%), malted soybean flour (20%–27.5%), and papaya fruit powder (10%–12.5%). In conclusion, the present approach can help in improve infants dietary quality of complementary foods by developing nutritionally enhanced red teff‐based porridge used for intervention of malnutrition.

## 1 INTRODUCTION

Many countries facing drastic public health challenges from malnutrition (Sonntag et al., 2014). Children's cannot grow and develop to their full potential because of malnutrition, around 45% of children deaths every year under age 5 are due to malnutrition (Black et al., 2013). Malnutrition, lack of dietary diversity, and poor quality diet are the largest risk factors responsible for the global burden of disease (Forouzanfar et al., 2015).

Global Nutrition Report (2016) presented that around 11% annual GDP losses in Asia and Africa were due to the malnutrition (low weight, poor child growth, and micronutrient deficiencies); this was greater than the loss experienced during the financial crisis of years 2008–2010.

FAO (2015) reported that of 667 million children (world population) under age 5 years, 129 million children's are stunted, 50 millions are wasted. Of 129 (data available) countries, 57 countries have serious levels of the malnutrition, and Ethiopia is one of them. In Ethiopia, 40.4% of children's were reported to be stunted and country ranked 117th of data available in 132 countries about children's stunting prevalence. In Ethiopia, 8.7% of children under age 5 are wasted and country ranked 98th position of 130 countries in wasting prevalence. Both the stunting and wasting rates are decreased in Ethiopia when compared to the previous but, still country has facing these problems. World health organization and governments of all the nations are hardly trying to eradicate the malnutrition problems by year 2030 (Global Nutrition Report, 2016).

The period from birth to 2 years of age is a “critical window” (Dewey, 2003), especially, childrens are in greatest risk of nutritional deficiency and growth retardation between 6 and 24 months of age (Shrimpton et al., 2001). Adequate nutrition during infancy and early childhood is a fundamental need to the comprehensive development (Michaelsen, Weaver, Branca, & Robertson, 2000). Around 6 months of age introduction of complementary foods along with sustained breastfeeding is required as mother's milk solely cannot provide adequate energy (Krebs, 2007). Complementary food helps in promote growth and provides healthy life (Dewey, 2003; Krebs, 2007), but such foods should be include adequate quantities of animal foods and vitamin A‐rich fruits, vegetables every day (WHO 2003). Where it is not possible, the use of fortified complementary foods and vitamin, mineral supplements is necessary to ensure nutritional adequacy (Nestel et al., 2003).

Micronutrient deficiency is known as “ hidden hunger” (World bank 2015), yet related issue in Ethiopia. To tackle the problem of malnutrition in underdeveloped countries, support of micronutrients and vitamin supplementation programs for young children has been taken place(Bhutta, salam&Das, 2013). Ethiopia has a long history of receiving food aid (Del Ninno, Dorosh, & Subbarao, 2007), yet the country's record of child malnourishment remains high (Global Nutrition Report, 2015) and poses a significant burden in economic and social development (UNICEF 2014). Major contributing factors of malnutrition among the infants and children in Ethiopia are poverty and low purchasing power of the family (Girma & Genebo, 2002; Herrador et al., 2014).

In Ethiopia, concentrated hot porridge called “*genfo”* is consumed by the mother and less viscous is offered to children (at the age of 6 months), it is prepared with barley, whole wheat flour and clarified butter (Mohammed, Seleshi, Nega, & Lee, 2016; Umeta, West, & Fufa, 2005). Complementary foods in developing countries are mostly comprised of cereals or starchy root crops which are provided in the form of porridge, it results in deficiencies of key micro‐ (Fe, Zn, Ca, vitamin B2, vitamin A, and vitamin C) and macro (protein)nutrients (Allen, 2006). Federal Democratic Republic of Ethiopia Ministry of Health reported that malnutrition in infant population of Ethiopia due to lack of adequate and balanced diet even though Ethiopia is producing variety of agricultural products, and childrens are the most affected social group by this problem (FDREMoH 2003).

To address these problems, in this study, teff, soybean, and papaya used as ingredient for developing formulated porridge. Red teff flour (source of Iron), malted soybean flour (source of protein), and papaya fruit powder (source of vitamin A) were easily available abundantly for the population in the study area. Objective of this study was to optimize the ratios of red teff flour, malted soybean flour, and papaya flour for better nutritional and sensory acceptability of developed porridge.

## 2 MATERIALS AND METHODS

### 2.1 Raw material collection

Red teff (DZ‐01 99 Variety) was collected from Debre Zeit Agricultural Research Center, soybean (Clerk 63k Variety) was obtained from Jimma Agricultural Research Center, and papaya fruits (Solo variety) were collected from Gojeb Horazion Plantation PLC. All the raw materials were carefully selected based on the availability in local region and for their best quality and productivity.

### 2.2 Raw materials processing

#### 2.2.1 Red Teff Flour (RTF) preparation

Red teff was manually cleaned by winnowing and removed chaffs, dust, and other impurities. After cleaning, ground to flour by miller (Heavy‐duty cutting mill, SM2000/695upm, Germany) and sieved through a 0.5 mm sieve and packed in polyethylene bags (AACC 2000).

#### 2.2.2 Malted Soybean Flour (MSF) preparation

Malted soybean flour was prepared using the method described by Tizazu et al. (2010). Soybean was cleaned properly using dry cleaning methods to remove foreign materials; cleaned soybean was soaked in excess of potable water for 18 hr at room temperature. The soaked water was drained off, and remained soybean seeds were washed with distilled water. Then the soybean seeds were spread in growth cabinet (model: RGX250) and maintained at temperature of 25°C and 95% of relative humidity for 48 hr. The germinated soybean seeds were allowed to dry for 24 hr in hot air oven at 50°C. The dried soybeans were milled into fine powder using a miller (Heavy‐duty cutting mill, SM2000/695upm, Germany) and sieved through a 0.5 mm sieve and packed in polyethylene bags until further work carried out.

#### 2.2.3 Papaya Fruit Powder (PFP) preparation

Uniform size, matured fruits were harvested carefully and brought to the laboratory from field. The fruits were washed with potable water; fruits were peeled using vegetable peeler (paring knife) and cut into half in lengthwise carefully, seeds were scooped out with spoon. Uniformly sliced flesh was dried using hot air oven at 60°C for 24 hr. Finally, the dried papaya fruit slices were ground into powder by mill (Heavy‐duty cutting mill, SM2000/695upm, Germany), sieved through 0.5 mm sieve, packed in amber‐colored plastic container, and stored in subdued light until further use (Padmapriya, 2013).

## 3 EXPERIMENTAL DESIGN

Total of eleven treatment combinations were generated using D‐Optimal Mixture design with three ingredients (RTF, MSF, and PFP) using Minitab version 16 statistical software. The percentages of lower, upper range of three ingredients include 55%–70% for RTF, 20%–30% for MSF, and 5%–15% for PFP. These ranges were set based on previously reported studies on complementary foods prepared from grains, legumes, vitamin‐rich plant foods, and WHO infant feeding guidelines (Black, Pahulu, & Dunn, 2009; FDREMoH 2006; Tortoe et al., 2014).

### 3.1 Porridge preparation

The composite flours were added together in proportion given by the design (Table 2), and they were mixed using an electrical blender for 3 min at 200 rpm. The mixed flour samples were packed and sealed in high‐density polyethylene bags. Porridges were prepared according to Onabanjo et al. (2008) from each of eleven composite flours. First, 300 g of previously prepared composite flour was mixed with 300 ml of water in small plastic bowl to make slurry and kept aside. In one stainless steel pan, 300 ml of distilled water was boiled, once water reached boiling point, the previously prepared slurry was mixed into the boiled water and allowed to cook for 15 min at 90°C with continuous stirring to avoid coagulation and sticking to the pan, after it was removed from the stove. The prepared porridge was used for analysis (proximate, mineral, antinutritional and sensory properties).

### 3.2 Data collection and analysis methods

#### 3.2.1 Proximate composition

Proximate composition of each individual ingredient flours and formulated porridges was determined in triplicate.

The moisture content of the sample was determined using hot air oven method; protein content was determined according to Kjeldahl method of crude protein analysis using conversion factor as 6.25. Crude fat was determined using Soxhlet extraction method; crude fiber was determined by the nonenzymatic gravimetric method, and ash content was determined by official methods (AOAC 2011) with method numbers 925.10, 979.09, 2003.06, 920.168, 923.03, respectively. The total percentage of carbohydrate content was determined by the difference method as reported by Onyeike, Olungwe, and Uwakwe (1995). Gross energy was calculated according to the method developed by Osborne and Voogt (1978).

### 3.3 Determination of β‐carotene

β‐carotene was determined according to the method of Sadler, Davis, and Dezman (1990). Two grams of sample was mixed with one gram of CaCl_2_.2H_2_O and 50 ml extraction solvent (50% hexane, 25% acetone, and 25% ethanol), and the mixture was shaken after every five‐minute intervals for 30 min, and the mixture was maintained at 4 ± 1°C in refrigerator. 15 ml of deionized water was added to the mixture and shaken after every five‐minute interval for additional 15 min by maintaining at 4 ± 1°C. After the extraction process, the organic phase which contains β‐carotene was separated from the water phase using a separation funnel and filtered using Whatman^®^ qualitative filter grade 1. The extraction procedure was carried out under subdued light to avoid degradation of carotenoids. β‐carotene was estimated from a standard curve (*R* ^2^ = .998) prepared by dissolving of stranded analytical grade β‐carotene in the same solvent combinations which used for sample extraction; absorption of samples was determined using double beam UV‐Vis spectrophotometer at 450 nm wavelength.

### 3.4 Mineral analyses

The concentration of Ca, Zn, and Fe in porridge samples was measured by atomic absorption spectrophotometer (Perkin‐Elmer, Model 3100, USA) according to the method of Hernández, Fraga, Jiménez, Jimenez, and Arias (2005) after dry ashing of 5 g of sample. The resulting white ash was weighed, dissolved in 3 ml of concentrated nitric acid, and diluted with deionized water in a 25‐ml calibrated flask, and this solution was used to determine Ca, Zn, and Fe. Standard stock solution of iron, zinc, and calcium was prepared by appropriate dilution of stranded pure metals. The samples and standards were atomized using air–acetylene as a source of energy for atomization (AACC 2000). For iron content, absorbance was measured at 248.3 nm, and iron was estimated from a standard calibration curve which was prepared from analytical grade iron wire. For zinc content, absorbance was measured at 213.8 nm, and zinc level was estimated from a standard calibration curve prepared from ZnO. Calcium content was measured at 422.7 nm absorbance.

### 3.5 Determination of antinutritional factors:

#### 3.5.1 Phytate

The method described by Vaintraub and Lapteva (1988) was used to phytate determination. Five gram of dried sample was weighed and extracted with 10 ml of 0.2N HCl for 1 hr at an ambient temperature and centrifuged at 1107 *g* for 30 min. Two milliliters of wade reagent was added to 3 ml of the supernatant sample solution, homogenized, and centrifuged at 1107 *g* for 10 min. The absorbance was measured at 500 nm in double beam UV‐Vis spectrophotometer. The phytate concentration was calculated from the difference between the absorbance of the blank (3 ml of 0.2N HCl + 2 ml of wade reagent) and that of assayed sample. The amount of phytic acid was calculated using phytic acid standard curve, and result was expressed as phytic acid in μg/g fresh weight.

#### 3.5.2 Condensed tannin

Maxson and Rooney (1972) reported method was used for condensed tannin determination. One gram of sample was added in a screw cap test tube, and 10 ml 1% HCl in methanol was mixed to it. The tubes were closed with lid and allowed to shake on mechanical shaker for 24 hr at room temperature. The mixture was centrifuged at 123 *g* for 5 min, and 1 ml supernatant was mixed with 5 ml of vanillin‐HCl reagent in another test tube and left aside for 20 min until the reaction is completed, and the absorbance was read at 500 nm using double beam UV‐Vis spectrophotometer. The amount of tannin was calculated using tannin standard curve (prepared using D‐catechin), and result was expressed as tannin in mg/g.

### 3.6 Sensory evaluation

Eleven formulated complementary porridge samples were prepared and subjected to sensory evaluation. The evaluation was carried out based on appearance, aroma, taste, mouthfeel, and overall acceptability using a five‐point hedonic scale, where 1 = dislike extremely, 2 = dislike moderately, 3 = neither like nor dislike, 4 = like moderately, and 5 = like extremely (Lim, 2011). Total of 50 untrained panelists (mothers who have babies age between 6 and 24 months) were randomly selected from Jimma Town. The consumer panelists were informed about the five‐point hedonic scale and its use prior to assessment. Freshly prepared porridge was served on white plate, arranged, and coded randomly. During the evaluation, panelists were instructed to palate clean with water between each sample testing.

### 3.7 Data analysis

The data were analyzed using Minitab version 16 software package. The statistical significance of the terms in the regression equations was examined by analysis of variance (ANOVA) for each response, and the significance test level was set at 5% (*p* *<* .05). Normal distribution of the data was done; the fitted models for all the parameters were generated. Graphical optimization was carried out to determine the optimum formula of RTF based porridge by substituting with different levels of MSF and PFP in terms of best nutrient composition and sensory attributes (Montgomery, 2012).

## 4 RESULTS AND DISCUSSION

### 4.1 Proximate composition

The moisture content of individual components and porridge ranged between 3.45 and 8.38%, 5.2 and 6.8%, respectively (Table 2). Moisture content did not show any significant difference between all possible interactions (Table 1). The highest moisture content (6.8%) was reported in the complementary food with 65% RTF, 20% MSF, and 15% PFP (FM1), and the lowest moisture content (5.2%) identified in the formulation with 65% RTF, 30% MSF, and 5% PFP (FM11). The moisture contents of the composite porridge increased with increase in RTF (55%–70%) and PFP (5%–15%) substitution by a range of 5.5%–6.6% and 5.2%–6.8%, consecutively (Elleuch et al., 2011; Haruna, Udobi, & Ndife, 2011). Study performed by Nair, Joglekar, and Sandhya (2013) reported in contrast to this study, which showed that with increasing soy flour in rice‐based complementary food; the moisture content of the product was increased from 1.87 to 4.08. This reverse trend in this study may be because of germination and conventional drying during sample preparation.

**Table 1:** Analysis of variance (ANOVA) *p*‐values of proximate composition, energy, β‐carotene, minerals (Fe, Ca, Zn), antinutritional factors, and sensory properties of complementary food prepared from RTF, MSF, and PFP

Source	MC	Ash	CFA	CF	CP	CHO	En	β‐car	Fe	Ca	Zn	Phy	Tan	App.	Aro	Taste	Mf	OA
Linear	0.599	0.000	0.002	0.001	0.001	0.000	0.023	0.003	0.000	0.000	0.009	0.011	0.007	0.005	0.053	0.009	0.013	0.000
Quadratic	0.983	0.000	0.014	0.004	0.001	0.001	0.027	0.015	0.000	0.000	0.024	0.043	0.005	0.002	0.057	0.027	0.044	0.001
A*B	0.806	0.015	0.008	0.047	0.001	0.000	0.994	0.826	0.000	0.011	0.308	0.011	0.009	0.004	0.684	0.273	0.725	0.001
A*C	0.955	0.000	0.597	0.005	0.014	0.077	0.008	0.004	0.000	0.001	0.016	0.313	0.022	0.015	0.048	0.027	0.012	0.000
B*C	0.972	0.001	0.004	0.001	0.001	0.000	0.032	0.011	0.000	0.000	0.008	0.042	0.021	0.007	0.015	0.007	0.031	0.008

A, red teff flour; B, malted soybean flour; C, papaya fruit powder; MC, moisture content; CFA, crude fat; CF, crude fiber; CP, crude protein; CHO, carbohydrate; En, gross energy; Fe, iron; Ca, calcium; Zn, zinc; Phy, phytate; Tan, tannin; App, appearance; Aro, aroma; Mf, mouthfeel and OAm, overall acceptability.

The ash content gives an indication of the mineral composition of food materials. In this study, ash values were found in the range of 3.19%–4.11% in the porridge. The lowest ash content (3.19%) corresponds to the sample containing 65% RTF, 20% MSF, and 15% PFP (FM1). The ash content of porridge was highly significantly (*p* < .01) affected by proportion of RTF, MSF, and PFP in the mix (Table 1) in linear, quadratic models between RTF with PFP, MSF with PFP and significantly different among RTF and soybean. The ash content of the blends was increased gradually with increasing proportion of soybean flour and supported by the claims of Akpapunam, Badifu, and Etokudo (1997). Low‐ash content was observed as proportion of PFP increased in the mixture. This could obviously due to the nonsignificant quantity of ash in PFP (0.60%) (Padmapriya, 2013). A study conducted by Kouakou et al. (2013) reported that the ash content of flour blends increased from 2.33% to 2.55% as the amount of soybean increases from 0% to 45% in the composite flour prepared from millet, maize, and soybean composite flour. Okoye, Nkwocha, and Ogbonnaya (2008) stated that ash contents of the blended products increased as the level of soybean flour ratio increased.

Fat is an important source of energy for infants and young children. Dietary fats function in the increase of palatability of food by absorbing and retaining flavors. The crude fat amount of the individual formulated components RTF, MSF, and PFP was 2.60%, 18.22%, and 0.16%, respectively, whereas the crude fat levels of formulated porridge were ranged from 5.27% to 7.44% (Table 2). There was a highly significant difference (*p* < .01) in the linear model; significant difference (*p* < .05) in the interaction of RTF with MSF and MSF with PFP (Table 1). There was no significant difference (*p* > .05) in the interaction between RTF with PFP. The fat content was low in FM1 (RTF 65%, MSF 20%, and PFP 15%) with percentage of 5.27% but higher in FM11 (RTF 65%, MSF 30%, and PFP 5%), the highest percentage crude fat (7.44%). As the amount of soybean flour increases from 20% to 30%, the fat content of the porridge was showed increment from 5.27 to 7.44; this is because of high amount of fat found in soybean.

**Table 2:** Measured proximate content (%) and energy (kcal/100 g), β‐carotene and minerals of red teff‐based formulated porridge and individual flours

Formulation	Components (%)			Proximate composition								Minerals (mg/100 g)		
	RTF	MSF	PAP	MC	Ash	CF	CFI	CP	CHO	Energy*	β‐car	Fe	Ca	Zn
FM1	65	20	15	6.8	3.19	5.27	2.51	12.55	69.68	376.30	4.916	12.29	160.62	4.05
FM2	55	30	15	5.5	3.99	7.13	3.45	19.84	60.08	383.88	5.737	9.38	169.471	4.31
FM3	70	25	5	5.8	3.87	6.27	3.07	15.61	65.39	380.40	0.245	34.86	270.26	5.44
FM4	65	22.5	12.5	6.3	3.23	5.67	2.37	13.86	68.57	380.70	2.816	13.76	177.95	4.53
FM5	60	27.5	12.5	5.7	3.55	6.47	2.93	17.65	63.70	383.61	3.626	9.85	182.62	4.81
FM6	70	20	10	6.6	3.25	5.36	2.33	13.57	68.88	378.09	1.420	29.24	198.23	4.84
FM7	67.5	25	7.5	6	3.61	6.20	2.77	15.32	66.10	381.48	0.608	25.64	235.22	5.36
FM8	65	27.5	7.5	5.6	3.70	6.69	3.13	19.70	61.18	383.70	0.744	21.74	245.44	5.38
FM9	65	25	10	6.1	3.35	6.14	2.60	16.20	65.62	382.51	2.052	16.97	204.8	5.24
FM10	67.5	22.5	10	6.2	3.34	5.74	2.49	14.44	67.79	380.62	1.746	22.4	201.49	5.09
FM11	65	30	5	5.2	4.11	7.44	3.60	24.22	55.43	385.56	0.351	27.58	293.57	5.58
RTF	100	0	0	8.10	3.31	2.60	3.20	12.26	70.54	354.57	0.193	38.00	179.30	5.23
MSF	0	100	0	3.45	5.56	18.22	5.73	43.63	23.41	432.14	0.424	9.88	264.80	8.29
PFP	0	0	100	8.39	0.63	0.16	1.93	0.88	88.01	357.01	33.510	0.94	28.70	0.32

FM, formulation; RTF, red teff flour; MSF, malted soybean flour; PFP, papaya fruit powder; MC, moisture content (%); CFA, crude fat (%); CFI, crude fiber (%); CP, crude protein (%); CHO, carbohydrate (%), and β‐car, β‐carotene (mg/g), *Energy in (Kilo calorie/100 g).

This result was similar with Ayo, Ayo, Popoola, Omosebi, and Joseph (2014) who reported the effect of malted soybean flour addition on the quality of the *acha*‐based bread; fat content was increased from 12.02% to 18.34% with an increase in the percentages (0%–50%) of malted soybean flour. In other reported study, the fat content was similarly increased from 0.5% to 2.4% in the composite breads produced from soybean flour substitution (Ndife, Abdulraheem, & Zakari, 2011). Soybean is an important fat source in complementary foods (Martin, Laswai, & Kulwa, 2010). As the MSF percent increase the crude fat content also raised than others formulations. This is due to the superior quality of MSF over RTF and PFP in terms of fat content (Emire & Buta, 2015). Teff is a staple food grain from Ethiopia that has a favorable fatty acid composition compared with other staple foods (Michaelsen et al., 2011).

The crude fibers of the formulated porridges varied from 2.33% to 3.60%. The crude fiber content of the RTF, MSF, PFP (individual flour samples) was 3.20%, 5.73%, and 1.93%, respectively, as shown in Table 2. The formulated complementary porridge crude fiber content showed highly significant difference in linear model, whereas a significant difference (*p* < .05) in the quadratic model (Table 1). There was significant difference in the crude fiber contents of all formulated porridges made from composite flours. The crude fiber content of the porridge was raised with an increase in the levels of MSF and RTF supplementation. The highest crude fiber content (3.6%) was determined in porridge from FM11 (RTF 65%, MSF 30%, and PFP 5%) where as the lowest is identified in the porridge from FM10 (RTF 67.5%, MSF 22.5%, PFP 10%). Similarly, Puranik (2012) concluded that foods containing maximum level of soybean flour have high content of crude fiber. The crude fiber content of formulated porridge was higher (1.37%–2.12%) than study report on bread–fruit–soybean food (Ijarotimi and Aroge 2005). Nwosu, Owuamanam, Omeire, and Eke (2014) also indicated that crude fiber content (5.18%) is more in malted soybean flour. The biscuits prepared with soybean–*acha* reported to accepted up to 30% added malted soybean with corresponding fiber content of 13.40% which is higher than crude fiber content of present study (Ayo et al., 2014). Red teff has a relatively high proportion of bran, which is high in fiber (Bultosa, 2007). Therefore, higher dietary fiber content is similarly expected with increased ratio of RTF. RTF and MSF used in this study have higher fiber content and contributed for higher fiber as their proportion increased in the formulation.

The protein content of formulated porridges was ranged from 12.55% to 24.22%, and protein contents in RTF, MSF, and PFP are 12.26%, 43.63%, and 0.88%, respectively (Table 2). The protein content of the porridge showed highly significant difference in linear model, quadratic model (*p* < .01). Highly significance variation was observed RTF with MSF, between MSF and PFP; significant difference among RTF and PFP (Table 1). The highest protein content (24.22%) was observed in complementary food from FM11 (RTF 65%, MSF 30%, and PFP 5%), where as lowest protein (12.55%) identified in porridge from RTF 65%, MSF 20%, and PFP 15% (FM1) (Table 2). There is an increase in the protein content formulated porridge because of MSF percent increase. Malomo, Ogunmoyela, and Kukoyi (2012) reported that protein content of germinated soybean rises around 9.4% of crude protein than ungerminated. The increase in protein content observed may be due to the synthesis of enzymes or compositional proportion changes followed by the degradation of other components (Nwosu et al., 2014; Ugwuona, 2009). Emmanuel‐Ikpeme, Ekpeyoung, and Igile (2012) reported the progressive increase in the protein value with increase in soybean addition in composite flours. In fact, legumes are rich sources of proteins ranged from 20% to 50%.

Mohammed, Mustafa, and Osman (2009) reported that the supplementation of wheat flour with teff showed reduction in protein content. Similarly, RTF is lower in protein as compared to MSF but, higher than PFP. Bultosa (2007), Baye (2014) reported that protein content ranged from 11.1% to 8.7% with mean of 10.4% in red teff. Teff's amino acid composition is well balanced with a relatively high concentration of lysine (a major limiting amino acid in cereals) found in teff. Similarly, compared to other cereals, higher contents of isoleucine, leucine, valine, tyrosine, threonine, methionine, phenylalanine, arginine, alanine, and histidine are found in teff.

Carbohydrates are an important source of energy in human diets comprising 40%–80% of total energy intake. In addition to energy, the carbohydrate contained foods are vehicles for important micronutrients and phytochemicals (FAO/WHO 1998). 70.54%, 23.41%, and 88.01% of the carbohydrate contents were identified in RTF, MSF, and PFP, respectively. The carbohydrate of formulated porridges ranged from 55.43% to 69.68%. The highest amount of carbohydrate content (69.68%) determined in FM1 (65%, 20%, 15% of RTF, MSF, and PFP, respectively) and the lowest determined from the FM11 (RTF 65%, MSF 30%, and PFP 5%) (Table 2). Carbohydrate decline trend was identified as the amount of MSF increased. There was a highly significant difference (*p* < .01) in the linear model of carbohydrate (Table 1).

Even though there was no significant difference in quadratic model of carbohydrate, there was an increment from 55.43% to 69.68% in carbohydrate content as the amount of PFP increased from 5% to 15%. The carbohydrate content of the porridge was enhanced as the ratio of PFP increased. This could be due to high amount of carbohydrate content found in PFP, and it is reported that dried PFP contains major amount of carbohydrates (Padmapriya, 2013). The opposite trend to the PFP was observed by the increase in MSF concentrations. Germination process of soybean degrades the carbohydrate contents; this may be due to an increase in respiration rate during germination that brings about the release of energy from the breakdown of carbon compounds (Malomo et al., 2012).

The carbohydrate content was increased from 60.08% to 68.86% in the formulated porridge as the ratio of RTF flour varies from 55% to 70%. This trend might be due to higher contents of carbohydrate in RTF. Red teff contains 80% of the complex carbohydrates and starch content of approximately 73% in total carbohydrate, which made teff as a starchy cereal (Baye, 2014).

The gross energy of RTF, MSF, PFP was 354.57, 432.14, and 357.01 kcal/100 g, respectively, while for the formulated complementary porridge gross energy varied from 376.30 to 385.56 kcal/100 g (Table 2). The highest gross energy of the porridge prepared from FM11 (RTF 65%, MSF 30%, PFP 5%), while the lowest was identified in the FM1 (RTF 65%, MSF 20%, PFP 15%). The amount of calories in the porridge showed highly significant difference (*p* < .01) in linear and quadratic model of RTF with MSF and MSF with PFP, while there was no significant difference (*p* < .05) between RTF and PFP (Table 1). The soy flour supplementation possessed significant effect on the gross energy of prepared porridge. The sources of high energy level in formulated complementary food were linked to the high carbohydrate content in the RTF, PFP; proteins and lipids contents in the MSF. The formulated complementary foods' energy value (376.30–385.56 kcal/100 g) is greater than the recommendation of FAO/WHO/UNU (2004), for an infant complementary food (under 1 year) in developing countries, is ranged from 200 to 300 kcal/day. The energy needs from complementary foods for infants with “average” breastmilk intake in developing countries (Brown, Dewey, Allen, Saadeh, & Nutrition, 1998) are approximately 200 kcal per day at 6–8 months of age, 300 kcal/day at age of 9–11 months, and 550 kcal per day at 12–23 months of age. In industrialized countries, these estimates are different (130, 310, and 580 kcal/day at 6–8, 9–11, and 12–23 months, respectively) because of differences in average breastfeeding practices. Among breastfed children in developing countries, average breastmilk energy intake is 413, 379, and 346 kcal/day at 6–8, 9–11, and 12–23 months, respectively (Brown et al., 1998).

### 4.2 β‐carotene Content

Beta carotene is the best known of the carotenoids due to its abundance in diet; it acts as an antioxidant, contains provitamin A activity (Gropper & Smith, 2012). The β‐carotene content of components was 0.193 mg/g in RTF, 0.424 mg/g MSF, and 33.510 mg/g PFP. The β‐carotene content of porridge sample varied between 0.245 mg/g and 5.737 mg/g (Table 2). The highest β‐carotene content observed in porridge prepared with FM2 (RTF 55%, MSF 30%, and PFP 15%) while lowest in FM3 (RTF 70%, MSF 25%, and PFP 5%). Highly significant difference (*p* < .01) was resulted in β‐carotene content of the formulated porridges in linear model, interaction between RTF with PFP and significantly different (*p* < .05) in quadratic model and MSF with PFP (Table 1). The increment of β‐carotene content was parallel increase in the proportion of PFP. This is due to the high β‐carotene content of PFP (Padmapriya, 2013). Recommended dietary allowances of vitamin A are around 300 μg/day (Institute of Medicine Food Nutrition Board, 2001). One of the malnutrition problems related to the micronutrients is vitamin A deficiency, using the PFP in porridge, this problem can solve.

### 4.3 Mineral contents (Iron, Calcium, and Zinc)

The iron contents of the formulated porridges were found in a range of 9.38–34.86 mg/100 g. Iron content of 38.00 mg/100 g in RTF, 9.88 mg/100 g in MSF, and 0.94 mg/100 g in PFP was determined (Table 2). The compositions of Fe in the formulation showed highly significance different (*p* < .01) both in linear, quadratic model, and in all possible interaction of RTF, MSF, and PFP (Table 1). The highest Fe content (34.86 mg/100 g) found in FM3 (RTF 70%, MSF 25%, and PFP 5%) where as lowest Fe content (9.38 mg/100 g) found in FM2 (RTF 55%, MSF 25%, and PFP 5%). The increase in RTF % in porridge directly increased the Fe content; it is because of red teff is a good source of iron (Abebe et al., 2007). The calcium contents of the formulated porridges were found in a range of 160.62–293.57 mg/100 g, the highest found in FM11 (RTF 65%, MSF 30%, and PFP 5%) where as lowest found in FM1 (RTF 65%, MSF 20%, and PFP 15%). 179.30 mg/100 g, 264.8 mg/100 g, 28.7 mg/100 g were the Ca found RTF, MSF, and PFP, respectively (Table 2). Calcium content was found to be highly significant in linear, quadratic model, and in the interaction of RTF with PFP (*p* < .01); significantly difference (*p* < .05) in the interaction of MSF and PFP (Table 1). As the soybean concentration increased the increase in Ca concentration reported in formulated soy‐based beverage it is because of soybean contains high amount of Ca then other two ingredients.

The zinc contents of the formulated porridges were found in a range of 4.05–5.58 mg/100 g; the highest Zn content found in the porridge prepared from FM11(RTF 65%, MSF 30%, and PFP 5%) while the lowest found in FM1(RTF 65%, MSF 20%, and PFP 15%). The Zn content in RTF, MSF, PFP individually observed that 5.23, 8.29, and 0.32 mg/100 g, respectively. Zinc content was found to be highly significant in linear model and in the interaction of MSF with PFP (*p* < .01); significantly difference (*p* < .05) in quadratic model and the interaction of RTF with PFP. Even though it was varied in the amount of 4.05–5.58 mg/100 g as the amount of MSF increased, it did not show significant difference in interaction of RTF with MSF.

High amount of Ca and Zn was observed in porridge when MSF percent increased where as Fe content was increased in the formulation as the proportion of RTF increased. This could be due to high‐iron content in red teff variety. As reported by Abebe et al. (2007), red teff is superior in iron and calcium content. Girma, Bultosa, and Bussa (2013) also indicated that red teff has high‐iron content (36.2 mg/100 g), low zinc (1.50 mg/100 g), and calcium (66.6 mg/100 g) contents. Higher mineral content (iron, calcium, and zinc) in this study found in porridges prepared from different formulations is due to higher concentration of calcium and zinc in the MSF and iron in RTF.

### 4.4 Antinutritional factors of porridge

The influence of primary ingredients RTF, MSF, and PFP on antinutritional factor of the porridge was analyzed using analysis of variance. The P‐values for models and combination products are summarized in Table 1.

The phytate content of soy‐supplemented composite flours containing RTF, MSF, and PFP is given in Table 3. The result showed a significant difference (*p* < .05) on the linear, quadratic model, in the interaction of RTF with MSF; MSF with PFP, and there was no significant difference (*p* < .05) in the interaction of RTF with PFP (Table 1). The amount of phytate content of porridge made from different composite flours ranged from 244.748 to 295.168 μg/g. The phytate amount of individual flours of RTF, MSF, and PFP was 295.168 μg/g, 163.866 μg/g, and 232.143 μg/g, respectively. The highest phytate content (295.168 μg/g) was determined in the porridge prepared from FM1 (RTF 65%, MSF 20%, PFP 5%) where as lowest phytate content (244.748 μg/g) present in FM11 (RTF 65%, MSF 30%, PFP 5%). Both the RTF and PFP concentrations are raised the phytate in complementary foods. Phytates bind to minerals like Ca, Fe, Mg, and Zn make them unavailable for bioabsorption (Nelson, Ferrara, & Storer, 1968).

**Table 3:** Antinutritional factors in prepared porridge from RTF, MSF, and PFP fruit powder and individual flours

Formulation	RTF	MSF	PFP	Phytate (μg/g)	Tannin (mg/g)
FM1	65	20	15	295.168	0.239
FM2	55	30	15	256.303	0.265
FM3	70	25	5	273.109	0.128
FM4	65	22.5	12.5	282.563	0.201
FM5	60	27.5	12.5	268.908	0.226
FM6	70	20	10	285.714	0.115
FM7	67.5	25	7.5	275.210	0.141
FM8	65	27.5	7.5	263.655	0.179
FM9	65	25	10	278.361	0.184
FM10	67.5	22.5	10	280.462	0.154
FM11	65	30	5	244.748	0.162
RTF	100	0	0	295.168	0.111
MSF	0	100	0	163.866	0.261
PFP	0	03	100	232.143	0.542

RTF, red teff flour; MSF, malted soybean flour; PFP, papaya fruit powder.

The levels of tannin content showed a highly significant (*p* < .01) effect on linear, quadratic model, and interaction between RTF with MSF and significantly different (*p* < .05) effect on interaction among RTF with PFP; MSF with PFP indicated in Table 1. The amount of tannin content of porridge made from different composite flours ranged from 0.115 to 0.265 mg/g as shown in Table 3. The tannin contents of individual flour RTF, MSF, PFP were 0.111 mg/g, 0.261 mg/g, and 0.542 mg/g, respectively. The highest tannin content is observed in porridge prepared from FM2 (RTF 55%, MSF 30%, PFP 15%) where as lowest is determined in FM6 (RTF 70%, MSF 20%, PFP 10%). As the MSF and PFP concentrations increased in complimentary food, the tannin concentration also increased. Amount of tannin in the porridge was increased as the amount of MSF increased. This result occurred because soybean contains high amount of tannin (Samuel, Otegbayo, & Alalade, 2012). Findings of the present study are in agreement with that of Maqbool, Aurangzeb, Habib, and Khan (1987) who concluded that tannin content of wheat roti is increased by the increasing the supplementation level of soy flour. PFP fruits also contain low amounts of antinutrients like tannin (10.16 mg/100 g of dry matter), phytate (3.29 mg/100 g of dry matter), and oxalate (1.89 mg/100 g of dry matter) (Onibon, Abulude, & Lawal, 2007). Both phytate and tannin content have shown a reduction when it is compared to the raw value. This might result from the different germination, soaking, and heat treatment during processing of raw materials. Phytate content of 682–1374 mg/100 g and tannin 16 mg/100 g were reported in teff on dry matter base (Baye, 2014).

### 4.5 Sensory evaluation

Sensory evaluation of porridge produced from RTF, MSF, and PFP at different mixture ratio is presented in Table 4. There was highly significant difference (*p* < .01) in linear model of taste in the interaction of MSF with PFP and significant difference in quadratic model in the interaction of RTF with PFP. There was no significant difference in the interaction of RTF with MSF (Table 1). It was also indicated that there was highly significant difference (*p* < .01) in the linear and quadratic model between RTF and MSF; MSF with PFP and significant difference (*p* < .05) in the interaction of RTF with PFP on appearance. The linear and quadratic models for aroma were not showed significant difference (*p* > .05) in the interaction between RTF with MSF, and it was only significantly different (*p* < .05) in the interaction of RTF with PFP and MSF with PFP. The linear, quadratic model for the mouthfeel in the interaction between RTF with PFP and MSF with PFP showed a significant difference (*p* < .05), but there was no significant difference (*p* > .05) between interaction of RTF with MSF. There was high significant difference (*p* < .01) in the linear and quadratic model and in all interactions of RTF, MSF, and PFP in overall acceptability of the complimentary food.

**Table 4:** Sensory quality of porridge formulated from RTF, MSF, and PFP

Formulation	RTF	MSF	PFP	Appearance	Aroma	Taste	Mouthfeel	OA
FM1	65	20	15	4.50	4.93	4.94	4.9	4.97
FM2	55	30	15	4.82	4.85	4.76	4.78	4.84
FM3	70	25	5	3.70	2.75	3.99	3.99	3.93
FM4	65	22.5	12.5	4.30	4.75	4.63	4.61	4.62
FM5	60	27.5	12.5	4.50	4.65	4.49	4.48	4.58
FM6	70	20	10	2.50	4.35	4.41	4.33	3.99
FM7	67.5	25	7.5	3.80	3.85	4.10	4.11	4.02
FM8	65	27.5	7.5	4.30	3.7	4.05	4.05	4.07
FM9	65	25	10	4.10	4.07	4.18	4.17	4.27
FM10	67.5	22.5	10	3.20	4.19	4.32	4.26	4.13
FM11	65	30	5	4.72	2.45	3.97	3.86	3.84

RTF, Red teff flour; MSF, malted soybean flour; PFP, papaya fruit flour; OA, overall acceptability.

**Table 5:** Regression models for nutritional and antinutritional compositions; mineral (Fe, Zn, Ca) and β‐carotene contents and sensory acceptability of porridge prepared

#	Porridge properties	Regression model	*R* ^2^ value
1	Moisture	7.976A − 7.625B + 11.844C + 8.447 + 1.935AC + 1.216BC	98.28
2	Ash	8.1A + 31.2B + 78.5C − 47.8AB − 111.4AC − 101BC	99.37
3	Fat	6.96A + 66.77B + 13.50C − 82.08AB − 10.85AC − 96.44BC	97.71
4	Crude fiber	6.1A + 45.6B + 94.9C − 63.6AB − 118.5AC + 168BC	98.82
5	Protein	39A + 704B − 303C − 1029AB + 570AC − 989BC	99.27
6	Carbohydrate	32.1A − 740B + 204C + 1214.2AB − 330.9AC + 1353BC	99.56
7	Gross energy (kcal/100 g)	45.9A + 456.9B − 273.3C + 1.5AB + 857.8AC + 588.9	98.01
8	β‐carotene	−0.9A − 0.9B + 346.3C + 18.3AB − 387AC − 312.8BC	99.34
9	Iron	279A + 951B + 408C − 2070AB − 1226AC − 1064	99.94
10	Calcium	355A + 1863B + 3213C − 2003AB − 4073AC − 8914BC	99.95
11	Zinc	1.70A − 11.52B − 98.71C + 36.23AB + 114.65AC + 137.67BC	98.48
12	Phytate	128A − 2106B + 1118C + 3764AB − 1063AC + 2595BC	98.33
13	Tannin	−0.997A − 4.334B + 9.718C + 10.371AB − 8.223AC − 8.395BC	98.96
14	Appearance	−24.7A − 109.5B + 227.4C + 268AB − 197.2AC − 241.9BC	97.73
15	Aroma	0.5A − 18.9B − 114.7C + 26.2AB + 158.6AC + 223.2BC	97.76
16	Taste	5.82A + 15.51B + 60.28C − 22.57AB − 56.99AC − 82.20BC	98.83
17	Mouthfeel	4.34A − 1.89B + 69.26c + 7.07AB − 73.12AC − 56.91BC	98.80
18	Overall acceptability	−3.6A − 46.8B105.5c + 98.6A − 105.6 AC − 56.2BC	99.58

A, RTF; B, MSF and C, PFP.

Lowest aroma, taste, mouthfeel, and overall acceptability ratings were given for formulations those had high amounts (30%) of MSF flours and low amount of PFP; in contrast, low proportions of MSF (20%) and high proportion of PFP (15%) had the best scores. Porridge with fairly balanced and intermediary proportions of RTF, low proportion of MSF, and a high amount of PFP (15%) had the highest aroma, taste, mouthfeel, and overall acceptability scores. But, appearance was lowest rating with those that had high amounts of RTF (70%), lowest amount MSF (20%) and PFP (5%) had low score.

A similar result was reported by Kanyago et al. (2007) with addition of soybean up to 25% gave the porridge with a good color, flavor, and taste. The overall result reported by Nair et al. (2013) also reveals that addition of soy flour at 20% was highly acceptable but the addition of soybean more than 25% the acceptability was decreased. There was enhancement in all the sensory characteristics in all the porridges except for appearance in porridge containing 67.5% RTF, 22.5% MSF, and 10% PFP. Sensory evaluation of the porridge showed that intermediate proportion of RTF, high amount of PFP in combination with low amounts of MSF (20%) resulted in a highly overall accepted (4.97) porridge formulation.

### 4.6 Optimization based on micro‐ and macronutrient composition

**Figure 1:**
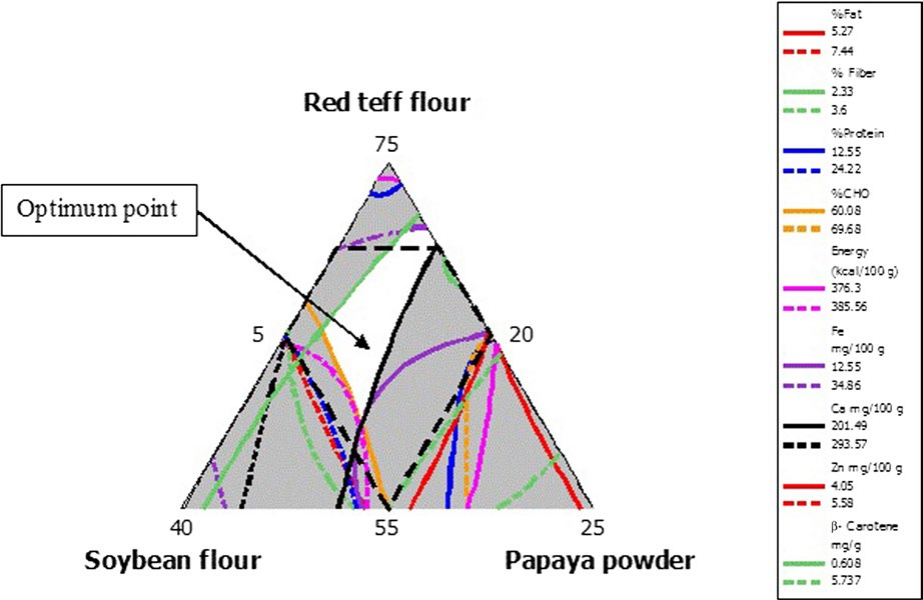
Overlaied contour plot of macro‐ and micro nutrients of the porridge

The optimum point includes all the parameters considered at optimum value (fat % and fiber%, protein%, carbohydrate%, gross energy (kcal/100 g), iron (mg/100 g), calcium (mg/100 g), zinc (mg/100 g), and β‐carotene (mg/g)) is indicated in Figure 1. The optimum point was found (determined by numerical optimization) in the porridge samples prepared within the range of RTF 60%–70%, MSF 20%–27.5%, and 10%–12.5% PFP. From the optimal value, it can be seen that the amount of MSF can be used from the lowest value to the maximum without affecting the nutritional content where as the optimum nutritional content was found at the maximum amount of MSF. Porridge prepared with this optimum point components the composition of porridge will contains 5.27%–7.44% fat, 2.33%–3.6% fiber, 12.55%–24.22% protein, 60.08%–69.68% carbohydrate, 376.3–385.56 kcal/100 g energy, 12.55–34.86 mg/100 g of Fe, 201.49–293.57 mg/100 g of Ca, 4.05–5.58 mg/100 g of Zn, and 0.608–5.737 mg/g of β‐carotene.

### 4.7 Optimization based on sensory evaluation

**Figure 2:**
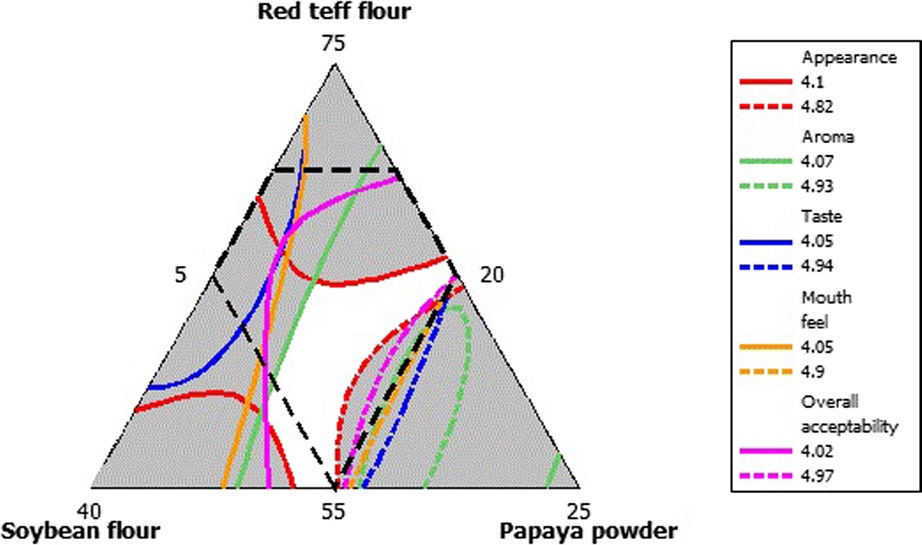
Overlaied contour plot of sensory acceptability of porridge

Although an optimal formation should maximize consumer acceptance, it is impossible to develop a product with all five sensory qualities that would satisfy consumers in most applications. The sweet point was obtained by placing a range of appearance, taste, aroma, mouthfeel, and overall acceptance (Figure 2). The optimum region in overlaid plot was where the criteria for all the five response variables (appearance, taste, aroma, mouthfeel, and overall acceptance) were satisfied and this region is found in the range of RTF 55%–65%, MSF 20%–30%, and PFP15%. With this range, the prepared porridge will show sensory acceptability of 4.1–4.82 appearance, 4.07–4.93 aroma, 4.05–4.49 taste, 4.05–4.9 mouthfeel, and 4.02–4.94 overall acceptability for porridge.

### 4.8 Overall optimal mixture compositions (Nutritional and Sensory)

**Figure 3:**
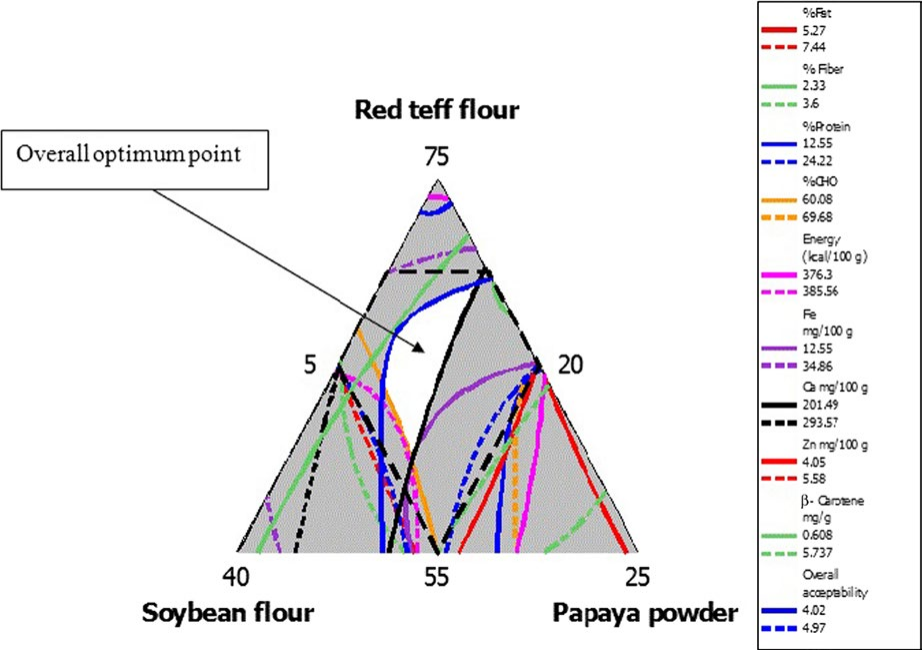
Overlaied contour plot for overall optimum points of fat, fiber, protein, carbohydrate, energy, iron, calcium, zinc, β‐carotene, and overall acceptance of porridge

In order to determine the optimum formulation, the regions of acceptability in the contour plot for protein, carbohydrate, fat, calorie, fiber, iron, calcium, zinc, and overall sensory attribute were superimposed. Superimposition contour plot was generated with most important parameters (protein %, carbohydrate%, fat%, energy (kcal/100 g), iron, zinc, calcium mg/100 g, and overall acceptance (received hedonic ratings)) to find out the optimum regions. The white region in the Figure 3 indicates that any point within this region represents an optimum combination of RTF, MSF, and PFP, which result in desirable attributes in both nutritional quality and sensory properties. The overall optimum values were found in a range of RTF 60%–70%, MSF 20%–27.5%, and PFP 10%–12.5%. Best fitted regression models and respective R2 values for nutritional and anti‐nutritional compositions; mineral, β‐carotene contents and sensory acceptability of porridge are presented in Table 5.

## 5 CONCLUSIONS

The porridges prepared with formulation of three basic components showed the moisture, ash, fat, crude fiber, protein, carbohydrate, energy, iron, calcium, zinc, β‐carotene, phytate, tannin, and overall acceptability were ranged between 5.2% and 6.8%, 3.19 and 4.11%, 5.36% and 7.44%, 2.49% and 3.6%, 12.55% and 24.22%, 55.43% and 69.68%, 386.56% and 383.88 kcal/100 g, 9.38 and 34.86 mg/100 g, 160.2 and 293.57 mg/100 g, 4.05 and 5.58 mg/100 g, 0.245 and 4.916 mg/g, 244.748 and 295.168 μg/g, 0.128 and 0.265 mg/g, 3.84 and 4.95 (on five‐point hedonic scale), respectively.

The result of this study indicated that the protein, fat and energy, mineral contents were significantly improved with increasing the proportion (20%–30%) of the MSF in the composite flour formula. β‐carotene and carbohydrate ratio of the product increased significantly with the increasing proportion of PFP powder. The phytate, tannin concentration in the porridge was increased as the levels of MSF and PFP increases to 30% and 15%, respectively. The sensory acceptability of almost all formulated complementary porridge scored better value in terms of appearance, aroma, taste, mouthfeel, and overall acceptability. Higher proportion of PFP powder in the formulation of porridge was showed good sensory acceptability. The RTF, MSF, and PFP resulted in a significant improvement in the porridge nutritional and sensory quality. The result revealed that RTF, MSF, and PFP have good potential source for providing protein, fiber, carbohydrate, energy, β‐carotene, and mineral‐rich complementary foods. Formulation of MSF, PFP containing RTF based porridge as complementary food produced a higher nutritional quality food with acceptable sensory characteristics when compared to the normal cereal‐based porridge nutritional quality of the region.

The overall optimum point was found in a range of RTF 60%–70%, MSF 20%–27.5%, and PFP 10%–12.5%. The formulation within the optimum may give porridge with fat 5.27%–7.44%, fiber 2.33%–3.6%, protein 12.55%–24.22%, carbohydrate 60.08%–69.68%, energy 376.3–385.56 kcal/100 g, iron 12.55–34.86 mg/100 g, calcium 201.49–293.57 mg/100 g, zinc 4.05–5.58 mg/100 g, β‐carotene 0.608–5.737 mg/g, and overall acceptance of 4.02–4.97(on five‐point hedonic scale).

## CONFLICT OF INTEREST

The authors declared that they have no conflict of interest.

## ETHICAL CONSIDERATION

This study was approved by Institutional Review Board of Jimma University.
